# α_1_-Acid Glycoprotein with Highly Fucosylated Glycans as a Potential Diagnostic Marker for Early Detection of Hepatobiliary and Pancreatic Cancers

**DOI:** 10.3390/diagnostics15010040

**Published:** 2024-12-27

**Authors:** Mizuki Endo, Shin Yazawa, Rie Sano, Takehiko Yokobori, Ken Shirabe, Hiroshi Saeki

**Affiliations:** 1 Department of General Surgical Science, Graduate School of Medicine, Gunma University, Maebashi 371-8511, Japan; m11201017@gunma-u.ac.jp (M.E.); kshirabe@gunma-u.ac.jp (K.S.); h-saeki@gunma-u.ac.jp (H.S.); 2 Department of Legal Medicine, Graduate School of Medicine, Gunma University, Maebashi 371-8511, Japan; sanorie@kumamoto-u.ac.jp; 3 Department of Innovative Cancer Immunotherapy, Graduate School of Medicine, Gunma University, Maebashi 371-8511, Japan; bori45@gunma-u.ac.jp

**Keywords:** α_1_-acid glycoprotein, fucosylated glycans, tumor biomarker, early diagnosis of cancer, sensitivity, combination assay

## Abstract

**Background**: Previously, we reported elevated levels of fucosylated α_1_-acid glycoprotein (fAGP) in plasma samples from patients with diverse types of cancers. Accordingly, fAGP was assumed to be a potential biomarker for the early detection of cancers. **Methods**: The fAGP level was retrospectively measured in preoperative plasma samples from 213 patients with either hepatic, biliary tract, or pancreatic cancer and was analyzed together with levels of six existing tumor markers determined as reference standards. **Results**: When the cutoff value was set at 25.45 U/μg, elevated levels of fAGP were significantly observed in cancer patients. The sensitivity, specificity, and accuracy for the detection of malignancy in these diseases were determined to be 70.79, 51.72, and 68.12, respectively. In contrast, all the tumor markers exhibited low sensitivity and accuracy, even though they commonly had extremely high (≥80%) specificity. Further, a significant number of patients in both early and advanced clinical stages were found to be false negative in these tumor makers but were found to be positive in the fAGP level. A dramatic improvement in the diagnosis by tumor markers in such patients with all clinical stages was found by the determination of the fAGP level. This indicated that fAGP could serve to correct false-negative diagnosis with tumor markers. **Conclusions**: It is believed that fAGP could be a relevant, unique, and highly sensitive biomarker for early diagnosis of hepatobiliary and pancreatic cancers.

## 1. Introduction

**Figure 1:**
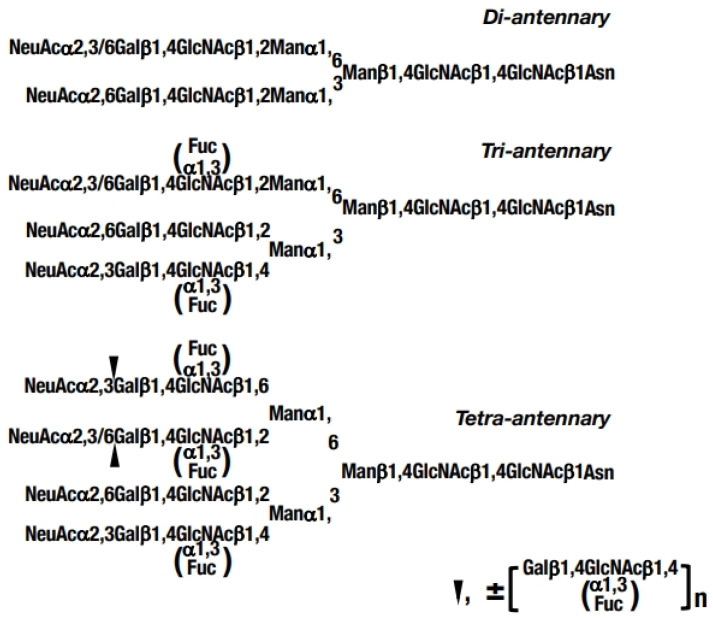
Branched *N*-linked glycans in α_1_-acid glycoprotein (AGP). One mole of AGP possesses 5 glycan chains including di-, tri-, and tetra-antennary glycan chains. Highly fucosylated AGP found in cancer patients were determined to be synthesized mainly on the tetra-antennary glycan chains accompanied by extremely elevated amounts of such glycan chains [22,23]. The presence of repeated lactosamine structures with Le^X^ structure (Galβ1,4[Fucα1,3]GlcNAc) in the tetra-antennary chains was predicted at the position of the black triangle in cancer patients. NeuAc, *N*-acetylneuraminic acid; Gal, d-galactose; GlcNAc, *N*-acetyl-d-glucosamine; Man, d-mannose; Fuc, l-fucose; and Asn, asparagine.

α_1_-Acid glycoprotein (AGP) is a major plasma glycoprotein with a molecular weight of 41–43 kD and is synthesized in the liver [1]. The potential physiological significance and functions of AGP have been investigated as the acute-phase protein with diverse immunomodulating effects and the molecule associated with systemic tissue injury, infection, inflammation responses, drug binding, and carrier as well as tumorigenicity [1,2,3,4,5,6,7,8,9]. Meanwhile, AGP has five highly sialylated and branched *N*-linked glycans, which consist of a combination of bi-, tri-, and tetra-antennary glycan chains (Figure 1). In the glycans of AGP, sialyl Le^X^ structures (NeuAcα2,3Galβ1,4[Fucα1,3]GlcNAcβR) are commonly present only in tri- and tetra-antennary chains with quantitative differences, and these glycans are recognized as having an important involvement in the inflammation response that occurs in a wide variety of diseases and physiological events including cancer [1,10,11,12,13,14,15,16,17,18].

Levels of AGP have been measured in plasma samples from cancer patients, and significantly elevated levels of AGP have been reported in them when compared with those in healthy controls and benign diseases resulting in the proposal as a tumor marker [8,19,20]. However, in our previous study using large numbers of samples from various cancer patients who had been followed for a long period after the operation, it was demonstrated that the level of AGP did not reflect patients’ clinical statuses or prognoses even though levels between patients and healthy controls were demonstrated to be significantly different [21]. In addition, with the aid of a crossed affinoimmunoelectrophoresis (CAIE) using Con A lectin and *Aleuria aurantia* lectin (AAL) to analyze branching degrees and extent of α1,3fucosylation in AGP glycans, respectively, together with anti-AGP antibody, AGP glycoforms were investigated. Accordingly, patients whose glycoforms contained highly fucosylated branched glycans for long periods of time after operation showed a poor prognosis. However, patients who had glycoforms without such glycans showed a good prognosis regardless of their clinical stages. The potential of AGP glycans expected as a novel tumor marker was further clarified by the following comprehensive analysis of AGP glycan structures in various cancer patients, in which a new MALDI-TOF-MS system was developed including a simple purification method of AGP and a rapid determination of primary structures of AGP glycans with the aid of the home-made software, AGPAS [22]. Then, detailed changes in fucosylated glycan structures in AGP that occurred in association with the presence of a malignant tumor were determined, for the first time, and the relative amount of α1,3fucosylated tri- and tetra-antennary glycans in all the glycans of AGP (FUCAGP) was found to be a promising marker to predict clinical outcomes along with patients’ responses to medication with various chemotherapies [23]. Recently, we developed an enzyme immunoassay system using anti-AGP antibody and AAL to establish a more rapid and precise method for the qualitative measurement of fucosylated AGP to analyze large numbers of samples at a time, in which an additional endeavor was conducted to improve sample handling and antibody preparation [24]. Further, using a new measurement value set up as a relative amount of fucosylated AGP (fAGP), this EIA method was applied for monitoring cancer patients who had received various chemotherapies. Importantly, no significant difference was found in performance values by FUCAGP and fAGP, respectively. Next, levels of fAGP were also measured in patients who had received immunotherapy with anti-PD-1 antibody after repeated treatment with unsuccessful chemotherapies [24,25], and in patients suffering from pancreatic intraductal papillary mucinous neoplasm (IPMN) using their preoperative sera [26]. It was of particular interest that the fAGP level appeared to be a reliable biomarker for not only predicting the clinical efficacy of immunotherapy treatment [25] but also for diagnosing the malignant potential of IPMN and predicting the malignant transformation of IPMN to IPMC [26]. Highly elevated expressions of fucosylated glycans in AGP, which occurred widely in association with the presence of malignant tumors, have also been reported recently in other studies [27,28,29].

Therefore, until now, the occurrence of cancer-associated changes in the fAGP level in diverse cancer types has been clarified together with the elucidation of the *FUT6* genes and α1,3fucosyltransferase as its key molecules although the total number of measurements was limited [21,22,23,24,25,26,30]. Furthermore, the recently developed simple assay method has made it possible to quickly examine a larger number of samples from preoperative cancer patients, which may have potential diagnostic applications in pancreatic cancer associated with previous studies on IPMNs [26].

In this study, the fAGP level was measured in preoperative samples from 213 patients with either hepatic, biliary tract, or pancreatic cancer in which no tumor markers with high diagnostic effect were present, in particular, in their early clinical stages. Then, the fAGP level was evaluated as a relevant biomarker for detecting malignant tumors along with measuring six existing tumor markers as reference standards.

## 2. Materials and Methods

### 2.1. Materials

Blood samples were preoperatively obtained from patients (*n* = 213) with either hepatic, biliary tract, or pancreatic cancer who were admitted at Gunma University Hospital between 2020 and 2023 with strict adherence to the set guidelines for informed consent together with approval from the Ethics Committee of Gunma University Graduate School of Medicine. In addition, approval was also obtained from the Gunma University Ethical Review Board for Medical Research Test Samples from Human Subjects (HS2023-042). The clinical stages of patients were determined by the TNM Classification of Malignant Tumours [31]. Blood samples were also obtained from volunteers as healthy controls (*n* = 135) whose standard values in blood tests were all within the baseline and with no history of chronic inflammation or hepatobiliary–pancreatic diseases, not just malignant tumors. Human serum AGP, bovine serum albumin, Tween 20, sodium metaperiodate, d-sorbitol, and papain were obtained from Sigma Aldrich (St. Louis, MO, USA). Peroxidase-conjugated ABC reagent, Vectastain Elite ABC standard kit, and biotinylated AAL were from Vecter Laboratories, Inc. (Burlingame, CA, USA). Anti-human AGP rabbit serum was from Dako (Carpinteria, CA, USA) and peroxidase-conjugated anti-human AGP monoclonal antibody was from Abcam (Cambridge, UK). KPL SureBlue TMB Microwell Peroxidase Substrate (1-Component) was obtained from Sera Care Life Sciences (Milford, MA, USA). Neuraminidase (*Arthrobacter ureafaciens*) was purchased from nacalai tesque (Kyoto, Japan). BlockAce was from DS Pharma Biomedical Co., Ltd. (Osaka, Japan). Levels of tumor markers were determined as CA19-9, CEA, and AFP by an ECLIA using Cobas system (Roche, Basel, Switzerland), PIVKA-II by a CLEIA using AIA-2000 (TOSOH, Kanagawa, Japan), DUPAN-2 by an EIA using AP-X (MINARIS MEDICAL, Tokyo, Japan), and SPan-1 by an IRMA using ARC950 (FUJIREBIO, Tokyo, Japan), respectively. Ant-Le^a^ and anti-Le^b^ monoclonal antibodies were from Ortho Clinical Diagnostics (Raritan, NJ, USA).

### 2.2. Determination of AGP and fAGP Levels

Levels of total AGP (ng/mL) and fAGP (U/μg AGP) in plasma samples were determined as described previously, which consisted of a sandwich-type ELISA for measuring the amount of total AGP (ng/mL) and an antibody/lectin EIA for measuring fAGP (U/mL) [24]. The samples were pretreated with neuraminidase to measure AGP and fAGP levels [24]. The cutoff value of fAGP was set at 25.45 U/μg in this study by calculating the mean + 2 S.D. of 135 healthy controls.

### 2.3. Determination of Lewis Blood Group Phenotypes

Lewis blood group phenotypes were determined using anti-Le^a^ and anti-Le^b^ antibodies by a hemagglutination test of erythrocytes pretreated with papain [32].

### 2.4. Determination of FUT3 and FUT6 Gene-Deficient Patients

Genotyping of the *Le* gene-dependent *FUT3* was performed as described previously [32] and by our newly developed STH-PAS method [33]. Genotyping was confined only to patients whose phenotypes were typed as Le (a−b−). The *FUT6* genotyping was also performed as described previously [34] and was confined only to patients whose fAGP levels were determined to be lower than 1.0 U/μg.

### 2.5. Statistical Analysis

The continuous valuables were analyzed by using an unpaired *t*-test. The significant differences between continuous variables were determined by using Student’s *t*-test or ANOVA. The relationship between variables of laboratory data and malignant or benign tumors was determined by using Pearson’s chi-square test or Fisher’s exact test. All statistical analyses were performed by using the JMP software, version 17.2.0 (SAS Institute Inc., Cary, NC, USA), and results were considered statistically significant at *p* < 0.05.

## 3. Results

### 3.1. Determination of Patients with FUT3 and FUT6 Gene Deficiency

Since *FUT3* and *FUT6* regulate CA19-9 and fucosylated AGP synthesis, respectively, it was confirmed, first, whether the present cohort consisting of 213 patients contained such gene-deficient individuals. Six patients were determined to possess lethal mutations (*le*/*le*) in the *FUT3* gene [32,33] and three of them were also found to have lethal mutations (*pf*/*pf*) in the *FUT6* gene [34]. Therefore, 207 patients were finally enrolled in this study.

### 3.2. The fAGP Level in Patients Measured According to Their Clinicopathological Features

Levels of fAGP were measured in 207 patients consisting of hepatic (*n* = 52), biliary tract (*n* = 90), and pancreas (*n* = 65) cancers. Of these, the tumors of 29 patients were found to be benign in the postoperative pathologic definitive diagnosis. The relationship between clinicopathological features and the fAGP level was examined in predefined subgroups of each index (Table 1). First, a statistically significant correlation was observed between the fAGP level and malignancy (*p* = 0.0496), which was independent of the organ-specificity in these three cancer types. Levels of fAGP seemed to elevate dependently with clinical stages of patients, but a significant correlation was observed only when clinical stages were classified into early (stages 0–1) and advanced (stages 2–4) stages (*p* = 0.0456). No such significances were found between other variables in clinical settings nor in laboratory data including CRP, as one of the inflammation markers, and six existing tumor markers were evaluated with their cutoff values.

### 3.3. Evaluation of the fAGP Level to Diagnose Malignancy

There were statistically significant differences in diagnosing malignant tumors based on fAGP (*p* = 0.0163) together with CA19-9 (*p* = 0.0041), SPan-1 (*p* = 0.0016), and CEA (*p* = 0.0328) (Table 2). fAGP had 70.79% sensitivity, 51.72% specificity, and 68.12% accuracy for the detection of malignancy. In contrast, all tumor markers exhibited a remarkably low sensitivity even though they had a high specificity of more than 80%. Among seven variables, the highest accuracy and sensitivity were obtained in fAGP, but a relatively low accuracy was determined in most tumor markers.

### 3.4. Differences in Positive Rates in fAGP and Tumor Markers Depending on Clinical

#### Stages and Respective Diseases

**Figure 2:**
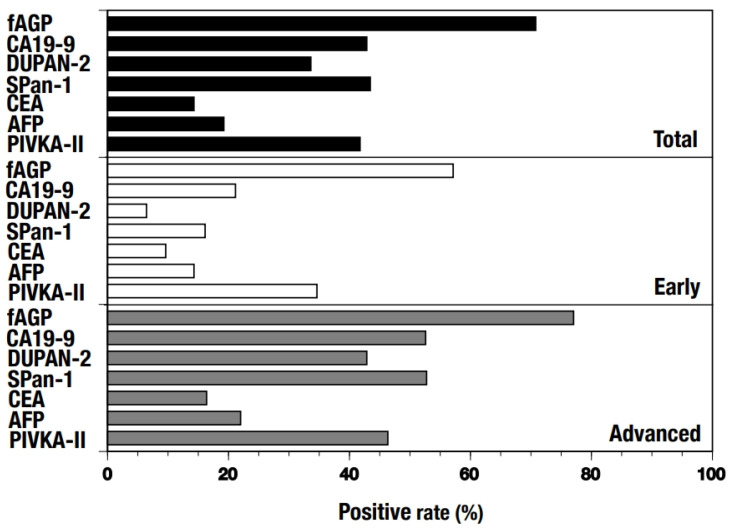
Positive rates of fAGP and respective tumor markers in plasma samples from patients with either hepatic, biliary tract, or pancreatic cancer. The total numbers of the patients examined were as follows: fAGP (178), CA19-9 (168), DUPAN-2 (122), SPan-1 (122), CEA (168), AFP (78), and PIVKA-II (67) (**top**). The positive rates were also determined depending on their clinical stages, such as in the early (stages 0–1) (**middle**) and advanced (stages 2–4) (**bottom**) stages, respectively.

**Figure 3:**
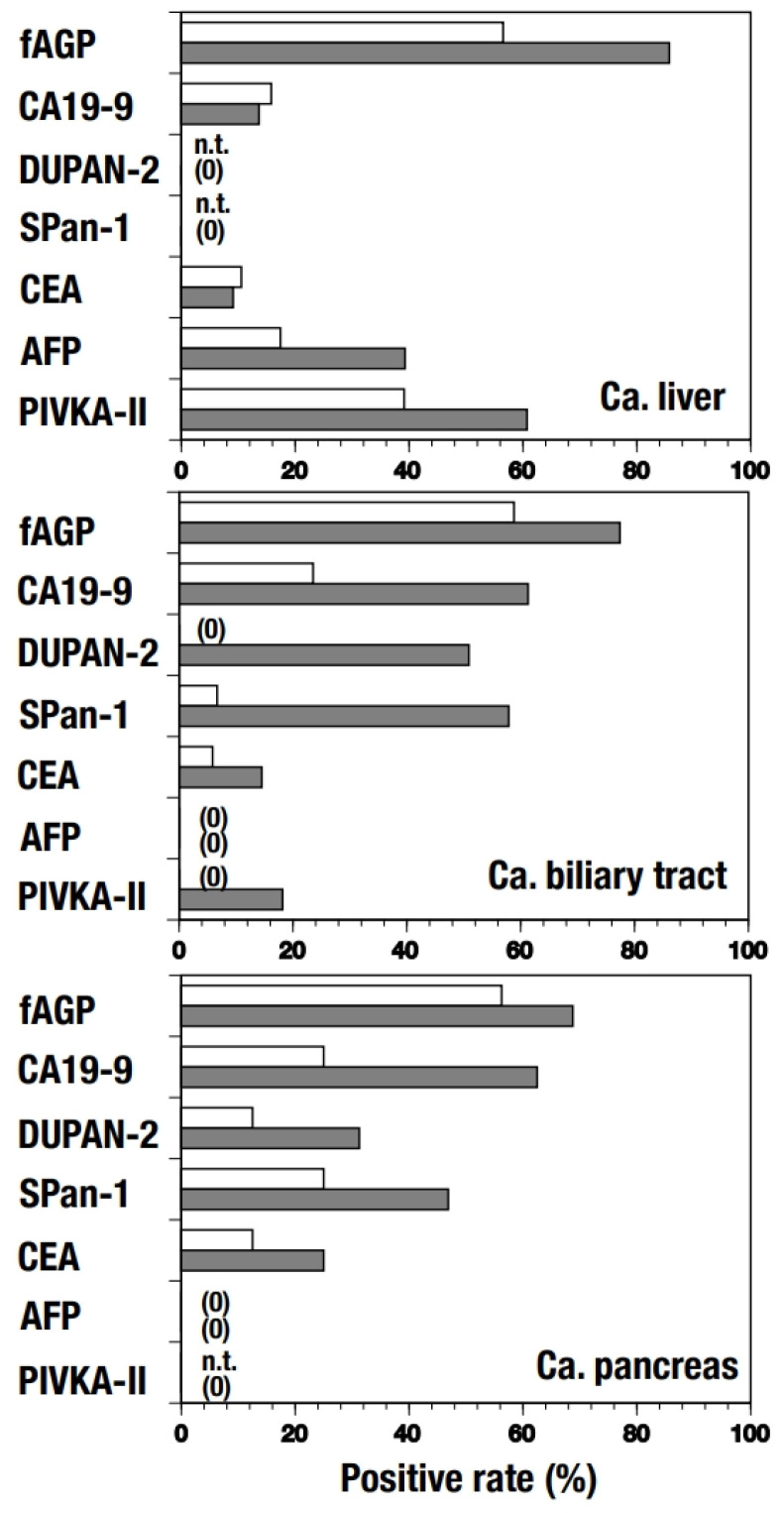
Positive rates of fAGP and respective tumor markers in plasma samples from patients with hepatic, biliary tract, and pancreatic cancer. White and gray bars (**top**, **middle**, and **bottom**) indicate corresponding positive rates in patients in the early (stages 0–1) and advanced (stages 2–4) stages, respectively. n.t. = not tested; (0) = no samples tested.

The positive rates of fAGP and the tumor markers were measured individually along with their clinical stages (Figure 2). The fAGP level showed the highest positive rate in every group, namely 70.79% in total, and 57.14% and 77.05% in early and advanced stages, respectively. In contrast, all the tumor markers but PIVKA-II were determined to have quite low positive rates in the early stage even though they showed increased positive rates, except CEA and AFP, in the advanced stage. Further, positive rates of fAGP and the tumor markers in patients with respective cancers were also measured depending on their clinical stages (Figure 3). In hepatic cancer, only fAGP (56.52%) and PIVKA-II (39.13%) showed higher positive rates in the early stage and significantly elevated rates in the advanced stage were also found in fAGP (85.71%) and PIVKA-II (60.71%). However, CA19-9 and CEA had very low positive rates in both stages. Whereas, in biliary tract cancer, only fAGP showed a high positive rate both in the early (58.82%) and advanced stages (77.42%), and three of the five tumor markers (CA19-9 = 61.29%, DUPAN-2 = 50.88%, and SPan-1 = 57.89%) had elevated values in the advanced stage despite their low positive rates in the early stage. In pancreas cancer, no high positive rate was observed in tumor markers but fAGP (56.25%) showed a high positive rate in the early stage. Fairly elevated positive rates in the advanced stage were observed in two tumor makers (CA19-9 = 62.50%, SPan-1 = 46.88%) as well as fAGP (68.75%).

### 3.5. Levels of fAGP Changed in Cancer Patients with Respective Diseases Depending on Some Clinical Settings and Laboratory Data

Levels of fAGP in cancer patients with respective diseases were also determined in connection with clinical settings and laboratory data (Table 3). Statistically significant differences in the fAGP level were found only in pancreas cancer (*p* = 0.0321) and CA19-9 (*p* = 0.0296). No significant difference was observed in the rest, although there were a few exceptions with results that were close to statistical significance.

### 3.6. Diagnostic Potential of fAGP in Cancer Patients with Respective Diseases Compared with That of Tumor Markers

The diagnostic potential of fAGP was analyzed and compared with that of six tumor markers for patients with respective cancer types (Table 4). The evaluation of fAGP and six tumor makers to diagnose malignancy indicated that CA19-9 (*p* = 0.0019) and SPan-1 (*p* = 0.0241) had a statistically significant difference in pancreas cancer but that no such significance was observed in the other factors nor in the other diseases with all factors even though there were a few exceptions that were close to being statistically significant. Then, the diagnostic values of fAGP and six tumor makers were also examined in the respective diseases. It was determined that fAGP exhibited the highest sensitivity in hepatic (72.55%), biliary tract (73.42%), and pancreatic (64.58%) cancers, respectively, and that most of the tumor makers showed low sensitivity far from these values. However, tumor makers showed considerably high specificity in every disease and 100% specificity was observed in three diseases with more than a couple of tumor markers. Even though 100% specificity was shown in hepatic cancer, fAGP was found to possess low levels in the other diseases. Conversely, the fAGP level exhibited the highest accuracy in hepatic (73.08%) and biliary tract (71.11%) cancer and high in pancreatic (60.00%) cancer. In contrast, the tumor markers showed quite low values in all diseases except CA19-9 (60.94%) in pancreatic cancer.

### 3.7. Complementary Uses of fAGP with Existing Tumor Markers for Diagnosis of Malignancy

**Figure 4:**
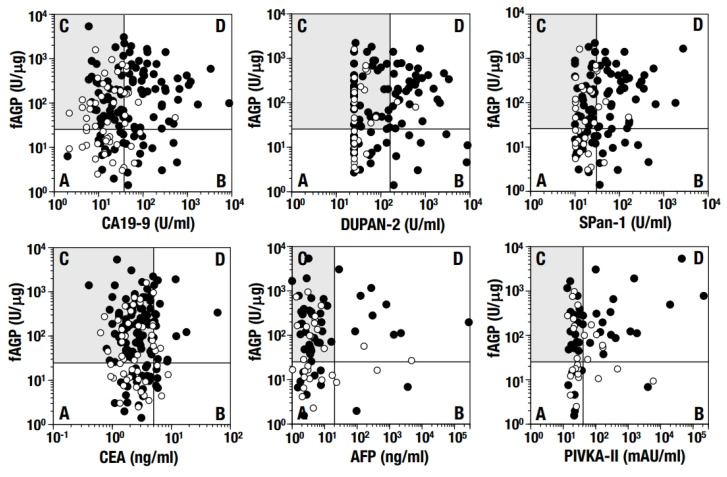
Diagnostic results of all patients were displayed as a set of levels of both respective tumor markers and fAGP, concurrently measured. Each symbol was plotted based on a set of levels of the tumor markers (horizontal axes) and fAGP (vertical axes). Four quadrants were defined by two axes lined at two cutoff values of the tumor markers (indicated) and fAGP (25.45 U/mg). Each quadrant includes patients who possess fAGP (−) and the tumor marker (−) (A), fAGP (−) and the tumor marker (+) (B), fAGP (+) and the tumor marker (−) (C, gray screen), and fAGP (+) and the tumor marker (+) (D), respectively. White and black circles indicate patients with early (stages 0–1) and advanced (stages 2–4) stages, respectively. Numbers of patients were as follows: in CA19-9/fAGP (168 with 52 early and 116 advanced stages), in DUPAN-2/fAGP (122 with 31 early and 91 advanced stages), in SPan-1/fAGP (122 with 31 early and 91 advanced stages), in CEA/fAGP (168 with 52 early and 116 advanced stages), in AFP/fAGP (78 with 28 early and 50 advanced stages), and in PIVKA-II/fAGP (67 with 26 early and 41 advanced stages).

The levels of fAGP and tumor markers measured in pairs were analyzed individually in both the early and advanced stages. To characterize the diagnosis with fAGP and each tumor marker, four groups of patients were set up according to levels either below or above the cutoff values of fAGP and the tumor markers, respectively (Figure 4). Namely, patients in groups A and B in Figure 4 consist of patients with false-negative fAGP results, whereas, patients in groups A and C consist of patients with false-negative tumor marker results, respectively. When focusing on patients diagnosed via CA19-9 and fAGP, 65 (group C in Figure 4, gray screen) of the 96 patients (group A plus group C) (67.71%) were determined to be negative based on the CA19-9 results but positive based on the fAGP results. Likewise, considerable numbers of false-negative patients determined via DUPAN-2 (64.20%), SPan-1 (62.32%), CEA (72.92%), AFP (69.84%), and PIVKA-II (61.54%) were diagnosed to have positive fAGP results. On the other hand, false-positive rates accompanied by these complementary uses of fAGP in respective tumor markers were found to still be very low when compared with their individual false-negative rates. The same complementary use of fAGP with existing tumor markers was further employed depending on the organ and clinical stage (Table 5). For the patients with false-negative results from respective tumor markers (group A plus group C in Figure 4), it was indicated that 50% or more were likely to be diagnosed as positive based on the fAGP results (group C in Figure 4) in all cases (*n* = 29) except four.

## 4. Discussion

Tumor markers have been used frequently in clinical management as a tool for diagnosing cancer, evaluating efficacies of chemotherapy, radiotherapy, and immunotherapy, and monitoring the prognosis of diseases such as recurrence and metastasis of cancer. A wide variety of molecules that are produced by cancer cells or normal cells in response to cancer have been found in tissues or body fluids including plasma along with the development of cutting-edge methods and techniques for their detection [35,36,37,38,39,40]. These molecules contain diverse proteins in glycosylated forms as glycoproteins and increased knowledge has extremely expanded the capability of not only further understanding the clinical importance of some glycoconjugates in glycoproteins but also applying certain glycoproteins as biomarkers for clinical purposes. Moreover, both the occurrence of tumor-associated alteration at some glycoconjugates in glycoproteins and the accumulated expression of such glycoproteins have also been reported in cancer [41,42,43,44,45]. Accordingly, a series of glycan-based tumor-associated antigens have been isolated and some of them have already been applied as tumor markers for monitoring and predicting patients’ prognoses during treatment interventions. Generally, tumor markers targeting carbohydrate antigens that commonly originate from tumors have a relatively high specificity to corresponding tumors with a certain degree of organ-specificity [46]. However, as is also well recognized, the diagnostic potential of these markers is often limited by low sensitivity, in particular, in patients at an early clinical stage or for screening purposes.

In our recent study, levels of fAGP were retrospectively analyzed in preoperative serum samples from large numbers of patients with intraductal papillary mucinous neoplasms (IPMNs) for the first time. It was demonstrated that the fAGP level changed significantly in association with malignancy and extremely elevated levels were found in patients with malignant IPMNs (IPMCs) but not in patients with benign IPMNs (IPMAs) [26]. Since no such changes were observed by using existing tumor markers such as CA19-9, DUPAN-2, SPan-1, or CEA, which were commonly used in these diseases, it was strongly suggested that fAGP could be used for preoperative diagnosis in hepatobiliary as well as pancreatic cancers.

Currently, a series of tumor markers are clinically available in patients with hepatobiliary and pancreatic cancers. In this study, the potential of fAGP and six existing tumor markers was evaluated for preoperative diagnosis of these cancer patients. Accordingly, the levels of fAGP were found to significantly distinguish patients with malignant tumors from those with benign diseases and were also observed to significantly elevate depending on their clinical stages. When the evaluation of diagnostic power was conducted using each cutoff value, levels of CA19-9, SPan-1, and CEA as well as the fAGP in patients with malignant tumors indicated differences from those in benign diseases but no such differences were present with the levels of DUPAN-2, AFP, or PIVKA-II. Whereas results from evaluations in respective cancers showed that significant differences were present only in pancreatic cancer diagnosed based on CA19-9 and SPan-1 levels. The diagnostic values of fAGP were accordingly determined as the sensitivity of 72.55%, 73.42%, and 64.58%, specificity of 100%, 54.55%, and 47.06%, and accuracy of 73.08%, 71.11%, and 60.00% in the hepatic, biliary tract, and pancreatic cancers, respectively. However, when compared with the diagnostic values of sensitivity and accuracy, the tumor markers showed extremely lower values than fAGP even though they possessed almost the same high specificity. Exceptionally, only CA19-9 was found to possess the highest accuracy in pancreatic cancer (60.94%). Therefore, fAGP was shown to possess diagnostic potential in all cancers regardless of their stages with the highest sensitivity together with the highest or higher accuracy depending on the diseases. However, among tumor markers, CA19-9 (pancreas and biliary tract), DUPAN-2 (biliary tract), SPan-1 (biliary tract and pancreas), and PIVKA-II (liver) were shown to possess relatively high positive rates with organ-dependency only in the advanced cancer stages.

CA19-9 is one of the most frequently applied tumor markers in patients with gastroenterological cancers, and the epitope was determined to possess sialyl Le^a^ structure (NeuAcα2,3Galβ1,3[Fucα1,4]GlcNAc) [47]. It has been demonstrated that CA19-9 has the potential for clinical utility as a serodiagnostic marker for patients with pancreas as well as biliary tract cancers [48,49]. However, it has been demonstrated that the antigen is hardly detectable in patients with early stages of cancer and that, in addition to the occurrence of false-positive results in some diseases, false-negative results are also unavoidable [50], which are caused by the absence of the *Le* gene-dependent Le enzyme in patients with *le*/*le* genotypes [32,33].

As described in our previous studies, the determination of genuine Lewis blood group types in cancer patients involves a chaotic situation, since Lewis blood group phenotypes are often typed in error due to the specificities of anti-Lewis antibodies [32]. Further, phenotyping of Lewis blood groups using patients’ erythrocytes raises false results because unexpected changes in phenotypes from Le (a−b+) and/or Le (a+b−) to Le (a−b−) occur frequently as found in the case of pregnancy [50]. To avoid serious mistyping and to determine genuine Lewis blood group types, it is necessary to conduct either the assay of the Le enzyme in saliva or the genotyping of the *Le* gene [32,33]. Recently, a novel method for the rapid identification of the *Le* gene was developed by means of the STH-PAS method using the *Le* gene-specific PCR product [33]. In this cohort, six patients were simply determined to be *le*/*le* homozygote cases by using this method, and they were excluded from this study because they could not synthesize Le antigens nor CA19-9. At the same time, three of these six patients were also determined to be *pf*/*pf* homozygote cases who could not synthesize fAGP as found previously [23,24,25,26,34].

Hitherto, the levels of fAGP in a wide variety of cancers, including esophagus, liver, pancreas, and colon [21], esophagus, stomach, lung and colon [22,23], esophagus, stomach, lung, and colon [24] and lung [25] cancers, have been demonstrated to be a reliable biomarker for not only predicting cancer prognosis and progression but also evaluating clinical outcomes of treatment with chemo- and immunotherapies. Even though there was a limitation on the number of patients examined and their analyses were performed differently by means of CAIE, MALDI-TOF-MS, and ELISA/EIA, exactly the same values for such diagnoses have been demonstrated during postoperative periods. Further, as described above, the fAGP level was applicable to diagnose the malignant potential of IPMNs of the pancreas and to predict malignant transformation using preoperative serum samples [26]. It was of particular interest that a combination of fAGP and PET/CT imaging offered the best diagnosis of IPMN, which resulted in further increases in the sensitivity, specificity, and accuracy of their diagnoses.

On the other hand, comprehensive analyses of AGP glycans in plasma from various cancer patients by using the MALDI-TOF-MS system indicated that the relative abundance of fucosylated glycans in AGP was significantly elevated in cancer patients unexceptionally and that such fucosylated glycans predominantly consisted of tri- and tetra-antennary glycan chains with mono- and difucosylated glycans (Figure 1) along with the occurrence of drastic changes in the glycan structures of the AGP molecule [22,23].

In the present study on patients with hepatobiliary and pancreatic cancer, it was of particular interest that fAGP was revealed to possess extremely high positive rates to detect malignancy in any case irrespective of the clinical stage when the cutoff value was set using the level obtained from healthy controls. It is worth noting that fAGP had high positive rates in patients in every case at the early stage (57.14%). Whereas, as shown in our previous studies [21,22,23,24,25,26], the tumor markers investigated in this study showed a poor diagnostic power, and their positive rates at the early stage were found to be quite low (less than 22%), except that of PIVKA-II (34.62%).

Moreover, it was revealed that the determination of fAGP levels provided further clinical benefit. Namely, large numbers of patients who had been diagnosed incorrectly as false-negative based on six tumor markers were determined to be positive based on fAGP. Therefore, fAGP seemed to possess preferential utility for not only early diagnosis of malignancy but also remarkable improvement in incorrect results from tumor makers regardless of their clinical stages, which must contribute to significantly increasing the diagnostic potential of existing tumor markers.

AGP has been investigated as an acute-phase protein that has physiological significance during an inflammation period and its pathological functions were also suggested previously [23,25,26]. In this study, it was suggested that levels of fAGP changed completely independently from those of existing tumor markers as demonstrated previously in patients with IPMNs [26]. Levels of CRP, one of the inflammation markers, also changed differently from those of fAGP and no statistically significant correlation was present between them. Additionally, levels of AGP in plasma as the inflammation maker were also observed to change differently from those of CRP.

It was noteworthy that α1,3fucoslyated glycans of AGP were synthesized in the liver by an action of the *FUT6* gene encoded α1,3fucosyltrasnferase, and that tumor-associated elevations of both fAGP levels and α1,3fucosyltrasnferase activities were widely determined in plasma samples from various cancer patients with no organ specificity [23,24,25,26,30]. Further, a significant correlation was found between the fAGP level and α1,3fucosyltrasnferase activity. In addition, individuals with *FUT6* deficiency were also observed to lack plasma α1,3fucosyltrasnferase activity and fucosylated AGP [34]. Whereas increased fucosylation in the AGP molecule occurred first from activation of the *FUT6* gene, which encoded α1,3fucosyltrasnferase, and then elevated expressions of fucosylated glycans on AGP with the aid of the enzyme accompanied by an increase synthesis of the AGP molecule in the liver were detected in plasma. The alteration of fucosylation in the AGP molecule consisting of a set of these processes induces not only in cancer patients but also in patients with noncancerous diseases with inflammation reactions [1,6] which results in uncertain diagnosis in some cases. However, it was revealed that fAGP possessed obvious utility when used alone as a new tumor marker with high sensitivity and wide usefulness or in combination with existing tumor markers as a complementary role to assist in reducing false-negative results.

Further, fAGP seemed to outweigh the disadvantage of the risk of causing false positive diagnoses as demonstrated in our previous studies [23,25,26]. In fact, in this study, it was demonstrated that more than 50% of all but except only a few patients with hepatobiliary or pancreatic cancer who were diagnosed to be negative by existing tumor markers were diagnosed to be positive based on fAGP, both in the early and advanced clinical stages. Therefore, false-negative results observed in existing tumor markers seemed to be significantly improved with the aid of measuring the fAGP level, which resulted in expanding the capability of precise diagnosis of malignancy. Further, as demonstrated in our previous studies [21,22,23,24,25,26], the fAGP level seems to change in association with clinical features of patients, and the measurement of the fAGP level in patients with various cancers including hepatic and pancreatic cancers during follow-up assessments for long periods after operation has revealed that fAGP is a clinical relevant biomarker of treatment intervention. Accordingly, it could be strongly proposed to apply fAGP not only for the early diagnosis of hepatobiliary and pancreatic cancers but also during follow-up assessments of patients who were grouped at the preoperative period as negative with existing tumor makers but positive with fAGP (patients in group C in Figure 4) in place of existing tumor markers. Most of the patients belonging to group C are now receiving follow-up treatment together with testing of additional samples to screen for different types of cancers.

In conclusion, currently, in clinical settings, existing tumor markers compared in this study are used in the diagnosis of hepatobiliary and pancreatic cancers. However, their diagnostic values of sensitivity or accuracy are not high, which causes inadequacy for early diagnosis of malignant tumors, notably, in patients with early clinical stages. In contrast, even though it seems somewhat less specific outside of hepatic cancer, fAGP has extremely high diagnostic values accompanied by diagnostic potential with the highest sensitivity for early diagnosis of these types of cancers including the early clinical stages. Further, complementary use of the fAGP level with existing tumor markers seemed to make a contribution to significantly improving their diagnostic potential both in the preoperative and postoperative periods. Therefore, fAGP appears to be a relevant and highly sensitive biomarker for early diagnosis of hepatobiliary and pancreatic cancers.

## Figures and Tables

**Table 1:** The levels of fAGP in patients that correlate with clinical settings and laboratory data.

Variables	*n*	fAGP (U/μg)	*p* Value
Clinical settings			
Age (years)			
≤74.9	125	215.3 ± 36.7	0.0998
>74.9	82	345.2 ± 79.3	
Gender			
Male	141	301.4 ± 48.8	0.1898
Female	66	192.7 ± 60.9	
Tumor location			
Liver	52	368.5 ± 121.7	
Biliary tract	90	241.2 ± 37.2	
Pancreas	65	220.7 ± 54.5	
Malignant tumor			
Negative	29	79.4 ± 32.0	0.0496
Positive	178	297.3 ± 44.2	
Clinical stage (I)			
0	4	41.9 ± 13.7	
1	52	176.8 ± 43.2	
2	73	370.3 ± 69.7	
3	33	345.1 ± 161.1	
4	15	337.7 ± 96.4	
Clinical stage (II)			
0–1	56	167.2 ± 40.4	0.0456
2–4	122 *	357.0 ± 61.0	
Laboratory data			
CRP (mg/dL)			
≤0.3	156	266.7 ± 49.0	0.9972
>0.3	51	267.0 ± 46.3	
CA19-9 (U/mL)			
≤37	120	226.5 ± 56.3	0.1762
>37	76	339.0 ± 54.0	
DUPAN-2 (U/mL)			
≤150	100	216.4 ± 41.4	0.4191
>150	45	274.1 ± 54.0	
SPan-1 (U/mL)			
≤30	90	202.5 ± 42.8	0.2175
>30	55	286.3 ± 50.8	
CEA (ng/mL)			
≤5	172	262.9 ± 43.3	0.6317
>5	24	322.2 ± 114.7	
AFP (ng/mL)			
≤20	68	287.6 ± 87.1	0.5003
>20	15	429.2 ± 206.7	
PIVKA-II (mAU/mL)			
≤40	41	220.5 ± 57.2	0.1138
>40	28	528.6 ± 217.80	

*n* = numbers tested. * one patient who had relapsed with residual pancreatic tumor was added. Cutoff values of tumor makers were set at 37.0 U/mL (CA19-9), 150 U/mL (DUPAN-2), 30 U/mL (SPan-1), 5.0 ng/mL (CEA), 20 ng/mL (AFP) and 40 mAU/mL (PIVKA-II), respectively.

**Table 2:** Characteristics of patients that correlate with the levels of fAGP and tumor markers and the evaluation of their diagnostic values, respectively.

		*n*				(%)	
Valuables	Total	Cancer	Non-Cancer	*p* Value	Sensitivity	Specificity	Accuracy
fAGP (U/μg)							
≤25.45	67	52	15	0.0163	70.79	51.72	68.12
>25.45	140	126	14				
CA19-9 (U/mL)							
≤37	120	96	24	0.0041	42.86	85.71	48.98
>37	76	72	4				
DUPAN-2 (U/mL)							
≤150	100	81	19	0.1231	33.61	82.61	41.38
>150	45	41	4				
SPan-1 (U/mL)							
≤30	90	69	21	0.0016	43.44	91.3	51.03
>30	55	53	2				
CEA (ng/mL)							
≤5	172	144	28	0.0328	14.29	100	26.53
>5	24	24	0				
AFP (ng/mL)							
≤20	68	63	5	0.5793	19.23	100	24.01
>20	15	15	0				
PIVKA-II (mAU/mL)							
≤40	41	39	2	0.5107	41.79	100	42.25
>40	28	28	0				

*n* = numbers tested.

**Table 3:** The levels of fAGP in patients with respective tumors that correlate with clinical settings and laboratory data.

		Liver			Biliary Tract			Pancreas	
Variables	*n*	fAGP (U/μg)	*p* Value	*n*	fAGP (U/μg)	*p* Value	*n*	fAGP (U/μg)	*p* Value
Malignant tumor									
Negative	1	10.65	0.6848	11	136.5 ± 81.8	0.2956	17	46.5 ± 11.3	0.0563
Positive	51	375.5 ± 123.9		79	255.8 ± 40.7		48	282.5 ± 71.8	
Clinical stage									
0–1	23	121.3 ± 39.2	0.0624	17	323.5 ± 114.3	0.3866	16	67.0 ± 17.6	0.0321
2–4	28	584.3 ± 217.3		62	237.2 ± 41.6		32	390.2 ± 102.5	
CRP (mg/dL)									
≤0.3	44	399.2 ± 142.7	0.5585	60	214.6 ± 48.4	0.3143	52	214.6 ± 61.7	0.8224
>0.3	8	199.2 ± 94.1		30	294.4 ± 55.1		13	245.5 ± 120.3	
CA19-9 (U/mL)									
≤37	36	395.3 ± 167.9	0.9045	45	176.7 ± 47.5	0.0826	39	128.2 ± 51.0	0.0296
>37	6	447.3 ± 298.9		45	300.7 ± 56.0		25	372.9 ± 112.2	
DUPAN-2 (U/mL)									
≤150	2	240.2 ± 153.2	-	49	191.4 ± 45.7	0.1175	49	240.4 ± 70.5	0.6090
>150	0	-		31	320.8 ± 73.2		14	170.6 ± 55.4	
SPan-1 (U/mL)									
≤30	2	240.2 ± 153.2	-	45	198.7 ± 50.1	0.2287	43	204.8 ± 73.1	0.6033
>30	0	-		35	296.7 ± 65.2		20	268.2 ± 82.7	
CEA (ng/mL)									
≤5	38	389.5 ± 159.0	0.7887	80	250.9 ± 41.4	0.4641	54	191.5 ± 52.6	0.1759
>5	4	528.0 ± 465.7		10	163.7 ± 42.3		10	398.4 ± 211.2	
AFP (ng/mL)									
≤20	37	343.8 ± 150.7	0.7540	25	240.6 ± 80.3	-	6	137.0 ± 72.6	-
>20	15	429.2 ± 206.7		0	-		0	-	
PIVKA-II (mAU/mL)									
≤40	26	179.1 ± 54.0	0.1207	13	335.7 ± 142.4	0.6257	2	11.2 ± 3.6	-
>40	26	557.9 ± 233.8		2	148.5 ± 29.7		0	-	

*n* = numbers tested.

**Table 4:** Characteristics of respective patients that correlate with the levels of fAGP and tumor markers and the evaluation of their diagnostic values, respectively.

		*n*				(%)	
Valuables	Total	Cancer	Non-Cancer	*p* Value	Sensitivity	Specificity	Accuracy
Liver							
fAGP (U/μg)							
≤25.45	15	14	1	0.2885	72.55	100	73.08
>25.45	37	37	0				
CA19-9 (U/mL)							
≤37	36	35	1	1	14.63	100	16.67
>37	6	6	0				
DUPAN-2 (U/mL)							
≤150	2	2	0	-	0.00	-	-
>150	0	0	0				
SPan-1 (U/mL)							
≤30	2	2	0	-	0.00	-	-
>30	0	0	0				
CEA (ng/mL)							
≤5	38	37	1	1	9.76	100	11.90
>5	4	4	0				
AFP (ng/mL)							
≤20	37	36	1	1	29.41	100	30.77
>20	15	0	15				
PIVKA-II (mAU/mL)							
≤40	26	25	1	1	50.98	100	51.92
>40	26	26	0				
Biliary tract							
fAGP (U/μg)							
≤25.45	27	21	6	0.0799	73.42	54.55	71.11
>25.45	63	58	5				
CA19-9 (U/mL)							
≤37	45	37	8	0.1076	53.16	72.73	55.56
>37	45	42	3				
DUPAN-2 (U/mL)							
≤150	49	43	6	0.4743	40.28	75.00	43.75
>150	31	29	2				
SPan-1 (U/mL)							
≤30	45	38	7	0.0735	47.22	87.50	51.25
>30	35	34	1				
CEA (ng/mL)							
≤5	80	69	11	0.6043	12.66	100	23.33
>5	10	10	0				
AFP (ng/mL)							
≤20	25	23	2	-	0	100	8.00
>20	0	0	0				
PIVKA-II (mAU/mL)							
≤40	13	12	1	1	14.29	100	20.00
>40	2	2	0				
Pancreas							
fAGP (U/μg)							
≤25.45	25	17	8	0.3965	64.58	47.06	60.00
>25.45	40	31	9				
CA19-9 (U/mL)							
≤37	39	24	15	0.0019	50.00	93.75	60.94
>37	25	24	1				
DUPAN-2 (U/mL)							
≤150	49	36	13	0.4862	25.00	86.67	39.68
>150	14	12	2				
SPan-1 (U/mL)							
≤30	43	29	14	0.0241	39.58	93.33	52.38
>30	20	19	1				
CEA (ng/mL)							
≤5	54	38	16	0.0559	20.83	100	40.63
>5	10	10	0				
AFP (ng/mL)							
≤20	6	4	2	-	0	100	33.33
>20	0	0	0				
PIVKA-II (mAU/mL)							
≤40	2	2	0	-	0	0	-
>40	0	0	0				

*n* = numbers tested.

**Table 5:** Relative rates in cancer patients who were diagnosed as negative with respetive tumor markers but positive with fAGP.

				(%) *			
Patient	Stage	CA19-9	DUPAN-2	SPan-1	CEA	AFP	PIVKA-II
Ca. Liver	E	62.50	-	-	64.71	57.89	50.00
	A	84.74	100	100	95.00	88.24	72.73
Ca. Biliary tract	E	53.85	60.00	57.14	62.50	25.00	33.30
	A	70.83	67.86	66.67	75.47	78.95	88.89
Ca. Pancreas	E	50.00	50.00	50.00	57.14	100	-
	A	58.33	68.18	64.71	70.83	33.33	0

* Nos. of fAGP (+)/nos. of tumor marker (−) × 100. E and A are early and advanced clinical stages, respectively. (-) = no sample tested.

## Data Availability

The datasets generated during and/or analyzed during the current study are available from the corresponding author on reasonable request.
